# SEEKR2: Versatile Multiscale Milestoning Utilizing the OpenMM Molecular Dynamics Engine

**DOI:** 10.1021/acs.jcim.2c00501

**Published:** 2022-06-27

**Authors:** Lane W. Votapka, Andrew M. Stokely, Anupam A. Ojha, Rommie E. Amaro

**Affiliations:** University of California, San Diego, 9500 Gilman Dr., La Jolla, California 92093, United States

## Abstract

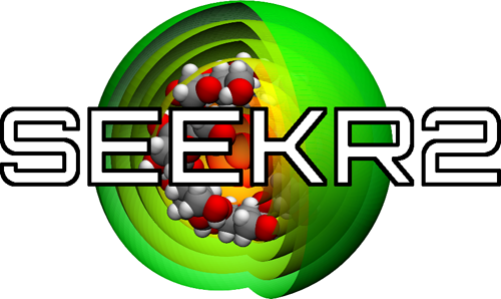

We present SEEKR2 (simulation-enabled estimation of kinetic rates version 2)—the latest iteration in the family of SEEKR programs for using multiscale simulation methods to computationally estimate the kinetics and thermodynamics of molecular processes, in particular, ligand-receptor binding. SEEKR2 generates equivalent, or improved, results compared to the earlier versions of SEEKR but with significant increases in speed and capabilities. SEEKR2 has also been built with greater ease of usability and with extensible features to enable future expansions of the method. Now, in addition to supporting simulations using NAMD, calculations may be run with the fast and extensible OpenMM simulation engine. The Brownian dynamics portion of the calculation has also been upgraded to Browndye 2. Furthermore, this version of SEEKR supports hydrogen mass repartitioning, which significantly reduces computational cost, while showing little, if any, loss of accuracy in the predicted kinetics.

## Introduction

### Background

The ability to computationally predict kinetic quantities, such as rate constants of reactions involving biomacromolecules, remains an active pursuit in computational and theoretical biophysics.^1−9^ Many approaches rely on sampling possible reaction pathways using simulation methods such as molecular dynamics (MD)^10−17^ and Brownian dynamics (BD);^18−22^ however, the main challenge arises from the need to sample many MD simulation trajectories to obtain accurate predictions for important kinetic quantities, such as the *k*_off_.^23^ At present, the amount of brute force MD simulations required to obtain kinetics of ligand binding and unbinding remains intractable for most applications involving biologically relevant targets. Therefore, many clever approaches to avoid the cost of brute force MD simulations use a wide variety of schemes to expand the temporal and spatial reach available to the computational biophysics community to predict kinetic quantities. We, and others, have summarized these approaches elsewhere.^24−43^

SEEKR is one method we developed to utilize both MD and BD approaches such that we may not only exploit MD when explicit solvent and full molecular flexibility are required but also exploit BD’s speed when semi-rigid body molecules and implicit solvents will suffice.^44−47^ SEEKR accomplishes this by partitioning the phase space of a system into smaller regions and then simulating trajectories within these regions using whichever is the most appropriate simulation approach, allowing each region to be simulated in parallel. The question of how to determine the best partitions of the MD and BD regions is still not completely understood, although we surmise that the BD region should extend beyond the first, and probably second, solvation shell to minimize inaccuracies caused by the implicit solvent. Within the solvation shells and the binding site itself, the explicit solvent and molecular flexibility of MD are likely required to obtain reasonable thermodynamic and kinetic quantities. In addition, by partitioning the phase space of molecular motion into smaller regions, one may ensure that events that are kinetically relevant but often rare are adequately observed and characterized. The statistics obtained from short simulations in each of these smaller regions are then stitched together using milestoning theory.^23,48−51^

SEEKR performed well in predicting ligand-receptor kinetics^44,52^ and was mostly successful in rank-ordering the affinity and residence times of a series of ligands binding to a receptor.^46,47^ The SEEKR approach was further augmented by utilizing a newer modification to milestoning theory, Markovian milestoning with Voronoi tessellations (MMVT).^46^ MMVT-SEEKR performed comparably well to the classical milestoning approach used in the earlier versions, with some added benefits, including an increase in accuracy for some quantities and decreased computational cost. All previous versions of SEEKR, including the MMVT version, used the NAMD simulation software package for MD.^53^

Here, we present SEEKR2, which uses the OpenMM simulation software suite^54^ for MD as an alternative to NAMD. OpenMM has enjoyed skyrocketing popularity among the scientific community because of its python interface, ease of extensibility, competitive performance on GPUs, and active development community. In addition, the design of OpenMM makes it relatively easy to develop independent plugins. Using SEEKR2, one may perform the MD portion of the SEEKR protocol in either OpenMM or NAMD, using either MMVT or the conventional milestoning method as developed by Elber and colleagues.^23,48,49^

SEEKR2 supports hydrogen mass repartitioning (HMR),^55^ allowing one to use a timestep of up to 4 fs. HMR works by repartitioning some of the mass of a heavy atom bonded to a hydrogen onto the hydrogen atom, enabling one to use a larger timestep without causing numerical instability in the simulation. Theory shows that HMR correctly produces thermodynamic quantities; however, its effect on kinetic quantities is unclear. A recent publication asserts that using HMR does not significantly affect kinetics in diffusing systems.^56^ We show in a later section that HMR produces reasonable kinetic results for systems within SEEKR2.

### Design and Implementation

#### The SEEKR2 OpenMM Plugin

The SEEKR2 OpenMM plugin design is based on OpenMM’s own layered architecture; it contains: (i) a Python interface layer for easy interactions with the user, (ii) CPU and GPU kernels, which include a number of integrators that implement the dynamics defined by MMVT or Elber milestoning, and (iii) a C++ API layer to connect the Python layer with the kernels.

A key aspect of the MMVT protocol is the definition of Voronoi cells. When a system reaches one of the boundaries of the cell, it collides against the boundary, and the identities and timescales of these collisions are logged for eventual analysis. SEEKR2 leverages the powerful custom mathematical expressions within the OpenMM package to define the locations of Voronoi cells and boundaries. By supplying a mathematical expression, the user can define the boundaries of a Voronoi cell, and when the system crosses it, the integrator logs the crossing information, the atomic positions and velocities are restored to the previous step, and velocities are reversed.

The mathematical expression for a given boundary defines a function, which can be any function of the system atomic positions. The functions for a cell (one function for each boundary) are defined by the user such that, when the system is inside the cell, the boundary function is negative. However, if the system ever crosses the boundary, then the value of the boundary function is positive for that system configuration. This makes the boundary a level set (or implicit surface). While more complicated than a simple Voronoi cell description, a level set description of a boundary is more general (able to define Voronoi cells and more), and SEEKR2 automates the most common milestone shapes that a user is likely to require. Such custom mathematical expressions make it straightforward to define Voronoi cells and boundaries in high-dimensional spaces.

An additional feature of the plugin allows users to optionally save OpenMM state objects whenever the boundary is crossed. These states can be used to analyze or visualize the locations of MMVT collisions or as a starting set of atomic positions/velocities for simulations in adjacent Voronoi cells. Users may also optionally set the plugin to compute MMVT rate matrices, incubation time vectors, and other quantities needed in post-processing analysis and then update them all to a file with each collision. There is negligible slowdown caused by this feature and can be used to compute convergence and other quantities “on-the-fly”.

#### The SEEKR2 Application Programming Interface (API)

We include additional scripts and programs (called the API) for preparation, running, and analysis of the simulations. For preparation of the SEEKR2 calculation, we include a utility that prepares the file tree and files for a SEEKR2 calculation, typically using concentric spherical milestones defined by the distance between the center of mass (COM) of atoms of a receptor binding site and the COM of a ligand molecule, as was used in our first MMVT SEEKR paper.^46^ For the convenient control of the SEEKR2 calculation, we include software that can be called with a single line that can run the simulations for any or all of the anchors and simulation regions. Last, we include an analysis package that will perform the milestoning calculations to obtain mean first passage times, rate constants, free energy profiles, and other quantities that are potentially desirable to obtain from a SEEKR2 calculation. Automated programs are also included to compute convergence.

In addition to these utilities, a number of example calculation scripts are included as well as comprehensive unit tests, documentation, and tutorials, all within a MolSSI cookiecutter, which we found at https://github.com/MolSSI/cookiecutter-cms.git. Within the API, it is possible to define a milestone as a type from a growing list of possible shapes. While all the milestones in this study are concentric spheres, it is also currently possible in SEEKR2 to connect milestones of non-concentric spherical shapes, planar milestones, milestones that are a linear, weighted function of distances, angles, and dihedral order parameters, and milestones that depend on the RMSD of a set of atoms to a reference structure. Simple examples of API usage may be found at https://seekr2.readthedocs.io/en/latest/api_examples.html.

## Results and Discussion

Benchmarking was performed to compare the performance of SEEKR2 against the original MMVT-SEEKR implementation in NAMD and a conventional OpenMM simulation of the same molecular system. Comparisons are listed in Table 1. For the trypsin-benzamidine system, the SEEKR2 OpenMM implementation performs almost 20 times faster than the NAMD-based MMVT-SEEKR code running on a GPU, which were re-run for the purposes of this study, and almost 6 times faster than the old MMVT-SEEKR running on a 68-core CPU node.^46^ The differences between the performances of the newer OpenMM approach and the use of NAMD for the MD portion of the SEEKR2 calculations are primarily caused by the implementation of the CUDA code to perform the milestoning procedure directly in an OpenMM plugin. In contrast, the NAMD approach currently utilizes a TCL-based interface, which negatively impacts the speed of milestoning calculations, even when the milestone crossings are only monitored every 10 timesteps. The NAMD3 program can run MD simulations on GPUs very efficiently, on-par with the speed of OpenMM on GPUs. However, at the time of this writing, NAMD3 has not implemented the TCL interface required to run milestoning, so we are currently limited to NAMD2, which, as can be seen from Table 1, does not run as efficiently on GPUs as OpenMM. Additionally, the OpenMM SEEKR2 plugin enables us to evaluate milestone crossings at every timestep while incurring little extra computational cost (as opposed to a default of milestone crossing evaluation every 10 timesteps when using NAMD). Compared to a conventional OpenMM simulation (without SEEKR2), only a ∼25% loss of speed was observed for these systems when the milestoning protocols were included in the SEEKR2 plugin.

**Table 1:** Trypsin-Benzamidine System Performance (∼23,000 Atoms)

MD engine	SEEKR version	computing resource	performance (ns/day)
NAMD2.13	MMVT SEEKR	Expanse V100 GPU (10 CPUs)	22
		Stampede CPU node (68 CPUs)	47
OpenMM7.5	SEEKR2	Expanse V100 GPU (1 CPU)	300
	conventional	Expanse V100 GPU (1 CPU)	416
	SEEKR2 with HMR	Expanse V100 GPU (1 CPU)	586

To ensure that SEEKR2 correctly replicated the rate constants as predicted in the original MMVT-SEEKR implementation,^46^ we repeated the host–guest and trypsin-benzamidine simulations in a nearly identical process (details listed in the Materials and Methods section) to obtain kinetic and thermodynamic quantities, which are reported in this section. In recent years, there has been an interest in studying the inhibitors of the Janus kinase (JAK)-signal transducer and activator of transcription (STAT) pathway, especially in cancer therapy. The JAK-STAT signaling pathway plays a critical role in regulating immune response, and any irregularities can lead to immune disorders. We demonstrate the ability of SEEKR2 to correctly estimate the extraordinarily long residence time of a novel ATP-competitive inhibitor of the JAK2-STAT5 signaling pathway.

Several of our previous papers have run SEEKR calculations on the trypsin-benzamidine system.^44,46^ In Table 2, the rate constants for those calculations are listed alongside the values computed using the SEEKR2 program. SEEKR2 obtains a *k*_off_ and a *k*_on_ that are within an order of magnitude of the experimentally measured quantities. Compared to previous versions of SEEKR, and compared to the experimental values, SEEKR2 without HMR obtains a *k*_off_ that is slightly too fast (990 ± 130 s^–1^ from SEEKR2 compared to experimental^57^ 600 ± 300 s^–1^). The *k*_on_ obtained by SEEKR2 is very close relative to experiment ((2.4 ± 0.2) × 10^7^ M^–1^ s^–1^ from SEEKR2 compared to the 2.9 × 10^7^ M^–1^ s^–1^ from experiment^57^). Finally, the Δ*G*_bind_ computed by SEEKR2 was off by a little more than 0.8 kcal/mol (−5.98 ± 0.09 kcal/mol from SEEKR2 compared to −6.71 ± 0.05 kcal/mol from the experiment^57^). Additional convergence analyses are reported in the Supporting Information for both the *k*_off_ (Figure S1) and the *k*_on_ (Figure S2). For the trypsin-benzamidine system, the total steered molecular dynamics (SMD) simulation time was 20 ns, the total MD MMVT simulation time was 5 μs, and 2 million BD trajectories were performed, in total. This is slightly more than the 4.4 μs of MD simulation time in our previous MMVT study.^46^ The simulations in this study were lengthened to obtain a round 500 ns per anchor, a simple choice in contrast with our previous MMVT study, which ended simulations as they satisfied a convergence metric resulting in arbitrary simulation lengths in each anchor. In addition, the SEEKR2 calculations were run with desolvation forces activated for the BD stage. This required us to adjust the outermost milestones of the trypsin-benzamidine system and even add a new milestone with a 16 Å radius. Without making this modification for SEEKR2, the desolvation forces and rigid body dynamics of BD prevented any reaction events from being observed, an issue that was not present for the MMVT-SEEKR version. Nevertheless, we believe that the desolvation forces add meaningful accuracy to the calculation, provided that the outermost milestone extends far enough into the solvent.

**Table 2:** Thermodynamics and Kinetics of Binding Results Computed for the Trypsin-Benzamidine System in Current and Previous Studies

trypsin/benzamidine	*k*_on_ (M^–1^ s^–1^)	*k*_off_ (s^–1^)	Δ*G*_bind_ (kcal/mol)
experimental^57^	2.9 × 10^7^	600 ± 300	–6.71 ± 0.05
SEEKR1 (2017) (ref)	(2.1 ± 0.3) × 10^7^	83 ± 14	–7.4 ± 0.1
MMVT SEEKR (2020) (ref)	(12.0 ± 0.5) × 10^7^	174 ± 9	–7.9 ± 0.04
SEEKR2 (2022)	(2.4 ± 0.2) × 10^7^	990 ± 130	–5.98 ± 0.09
SEEKR2 HMR (2022)	(8.6 ± 0.7) × 10^6^	310 ± 30	–6.06 ± 0.08

Results for the trypsin-benzamidine system when using HMR were fairly close to non-HMR and experimental values, yielding a *k*_off_ of 310 ± 30 s^–1^, a *k*_on_ of (8.6 ± 0.7) × 10^6^ M^–1^ s^–1^, and a Δ*G*_bind_ of −6.06 ± 0.08 kcal/mol. These results indicate that HMR is able to compute similar binding kinetics at half the cost of conventional MD (Table 1).

**Figure 1:**
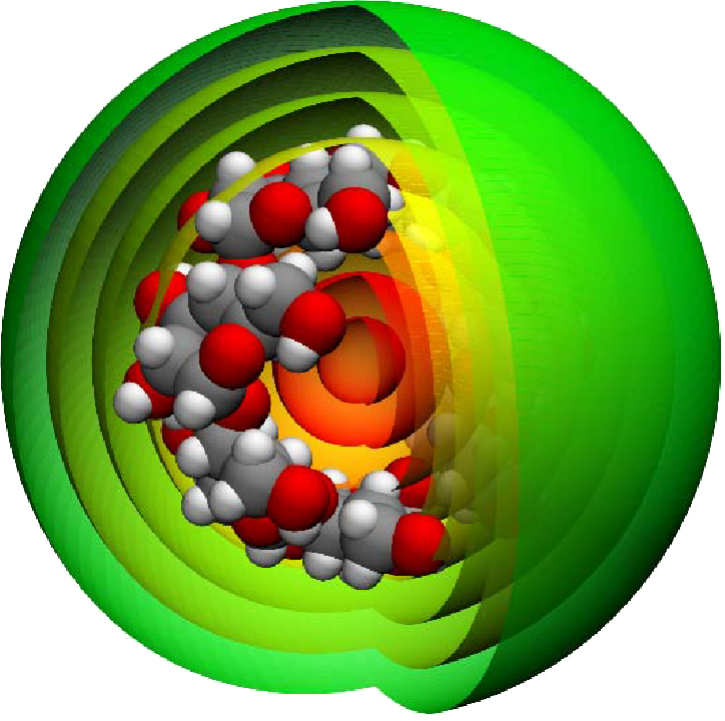
The space surrounding the β-cyclodextrin molecule is divided into 12 concentric spherical Voronoi tessellations (eight of the innermost ones are shown here), with boundaries that exist at 1 Å increments from 1 to 13 Å. OpenMM is used to run MMVT using MD within each of these cells, and trajectories collide against the boundaries between each cell, giving the transition times and statistics, which are analyzed with milestoning theory. This image was generated using VMD.^65^

**Figure 2:**
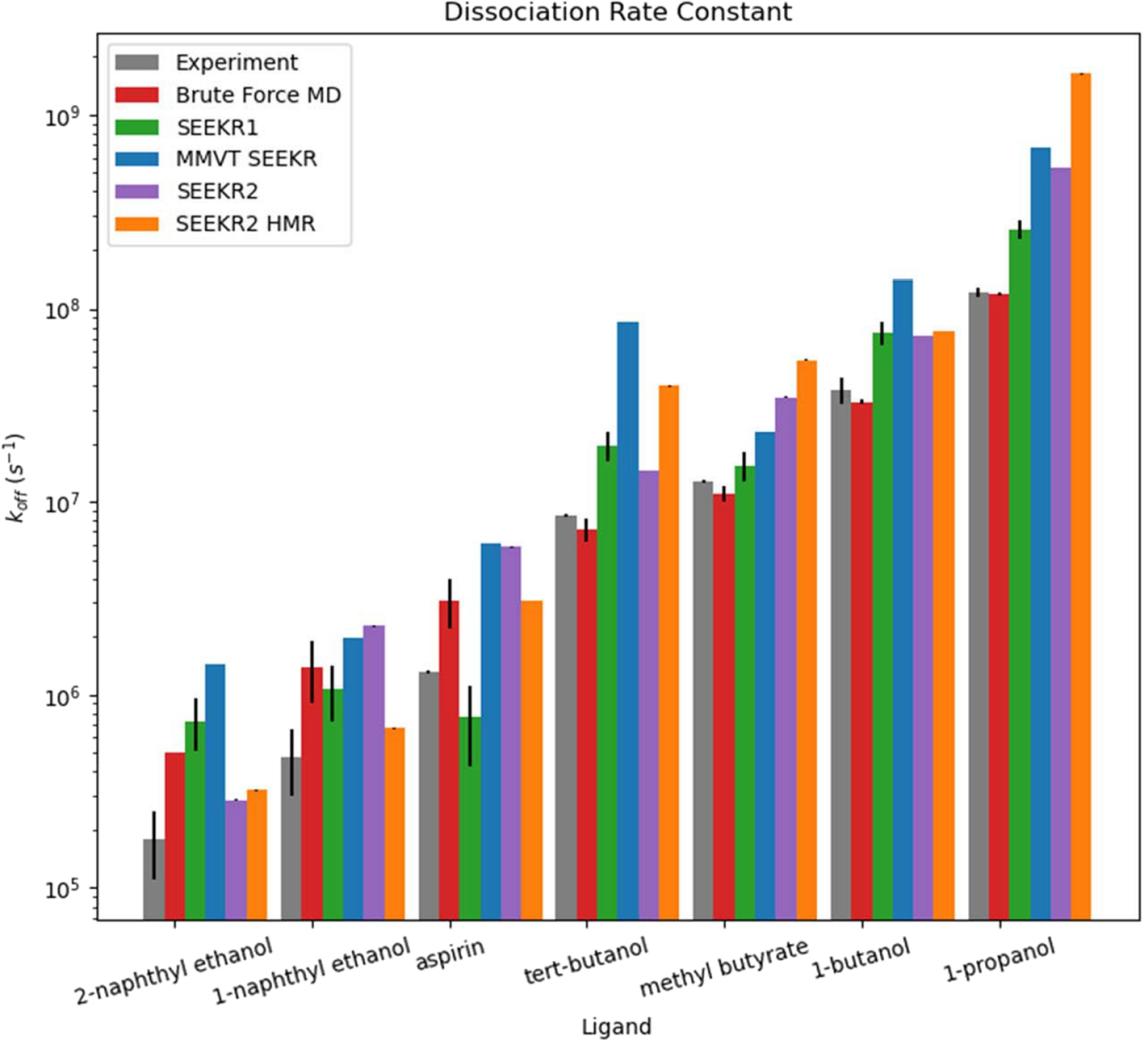
The *k*_off_s of each of the “guest” molecules to dissociate from the “host” molecule are listed. The “Experimental”, “Brute Force MD”, “SEEKR1”, and “MMVT SEEKR” results were generated in previous studies by others or us. The results labeled “SEEKR2” were generated in this study. SEEKR2 performs comparably or better than brute force MD and other computational methods. SEEKR2 is also the only method (aside from brute force MD) that correctly ranks the “guest” compounds by *k*_off_ according to the experiment. Error bars are present for the SEEKR2 data, but they are sometimes too small to see in this figure.

**Figure 3:**
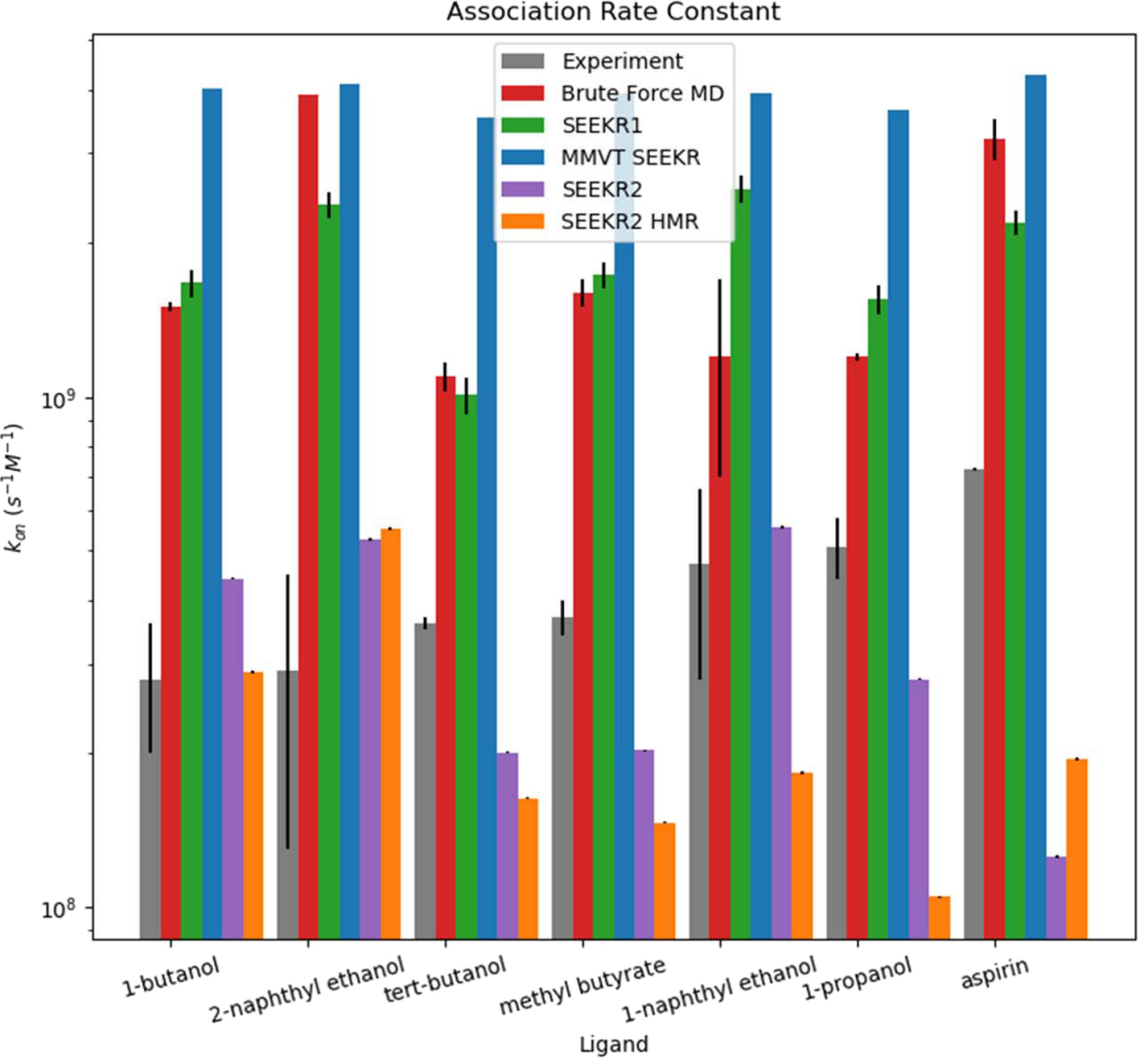
The *k*_on_s of each “guest” compound as they bind to the “host” molecule are shown in order of increasing experimentally measured *k*_on_. Previous studies had produced the “Experimental”, “Brute Force MD”, “SEEKR1”, and “MMVT SEEKR” results. The results of this study only produced the results labeled “SEEKR2”. SEEKR2 performs the best of all methods for estimating absolute *k*_on_ values. No methods were able to correctly rank *k*_on_s. This is likely due to the very small differences between experimentally measured *k*_on_s. Error bars are present for the SEEKR2 data, but they are sometimes too small to see in this figure.

**Figure 4:**
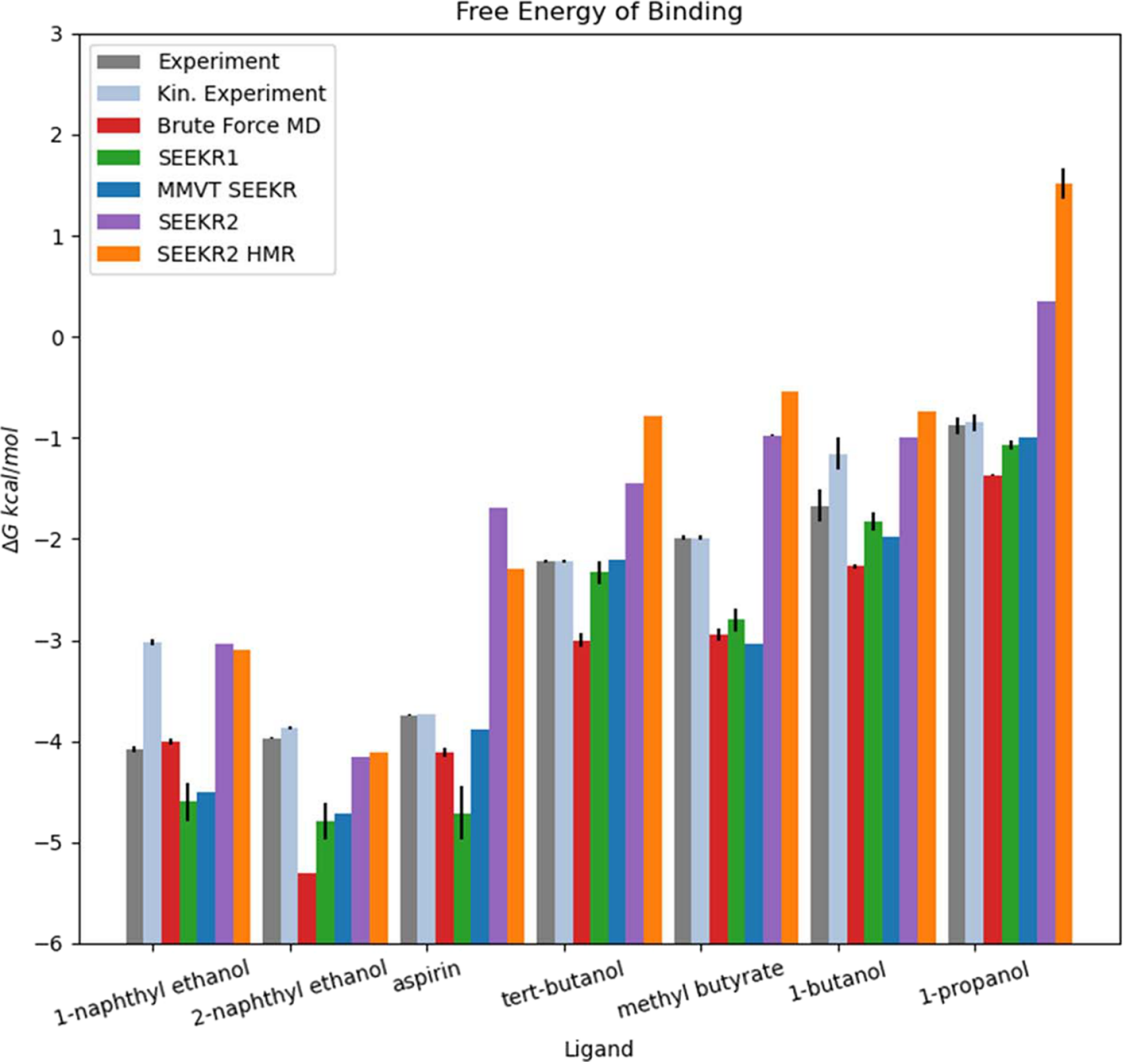
The Δ*G*_bind_ of each “guest” compound when binding to the “host” molecule ranked from lowest to highest Δ*G*_bind_. The quantities marked as “Experiment”, “Kin. Experiment”, “Brute Force MD”, “SEEKR1”, and “MMVT SEEKR” were published in previous studies. Only the quantities labeled “SEEKR2” were generated in this study. Note that two experimental values are listed—one where the Δ*G*_bind_ was measured directly, and one where Δ*G*_bind_ was computed from the experimentally measured *k*_on_ and *k*_off_. SEEKR2 correctly predicts compound ranking, with the exception of the first two compounds, which have very similar Δ*G*_bind_. Error bars are present for the SEEKR2 data, but they are sometimes too small to see in this figure.

In addition to the trypsin-benzamidine system, previous SEEKR studies have focused on computing the kinetics of a so-called “host–guest” model system, composed of the β-cyclodextrin and a series of small organic molecules.^47,58^ Using SEEKR2, as we did with MMVT SEEKR, we divided the space surrounding the β-cyclodextrin into 12 concentric spherical Voronoi cells (Figure 1) and recomputed *k*_off_ (Figure 2), *k*_on_ (Figure 3), and Δ*G*_bind_ values (Figure 4) for the seven ligands mentioned in previous publications, both with and without HMR.^46,47,58^ SEEKR2, both with and without HMR, now correctly ranks the compounds by *k*_off_. SEEKR2 also represents a substantial improvement in the calculations of absolute *k*_on_s for the host–guest system, although SEEKR2 does not correctly rank *k*_on_s for all seven host–guest systems, which is difficult for any method since the host–guest *k*_on_s differ by magnitudes that are relatively small compared to experimental margins of error. The Δ*G*_bind_ values were computed with fairly similar accuracy to previous calculations, with the exception of aspirin, which showed an anomalous Δ*G*_bind_ value. It is likely that the noise seen in the *k*_on_ and Δ*G*_bind_ calculations is primarily caused by the concentric spherical milestone shapes used in this study, which may not adequately approximate isosurfaces of the committor function for the host–guest system, as would be produced by exact milestoning theory.^23^ Additional milestone shapes have only been recently implemented in SEEKR2, which will allow us to investigate whether other types of milestones will improve the accuracy of *k*_on_ calculations for the host–guest system. For each individual host–guest system, the cost of the SMD simulations to generate a starting structure was 110 ns, and the MD MMVT ran for 700 ns each as well as 110,000 BD trajectories per guest molecule. This is slightly longer than the ∼560 ns total MD of our previous MMVT study.^46^ The reason for this is similar to the trypsin-benzamidine system above; the host–guest simulations in our previous study were halted after arbitrary times based on the satisfaction of a convergence metric. In this study, we elected to extend the simulations to 50 ns per anchor, for all host–guest systems, to simplify and standardize the calculation. As with trypsin-benzamidine, HMR was able to successfully predict binding kinetics for the host–guest systems at a reduced computational cost. The HMR-predicted kinetics were close to experimental values, albeit somewhat different from the results obtained using non-HMR simulations, although within experimental error and without sacrificing correct rankings, in the case of host–guest *k*_off_s. These results further demonstrate the utility of HMR to predict binding kinetics with associated cost savings using SEEKR2.

**Figure 5:**
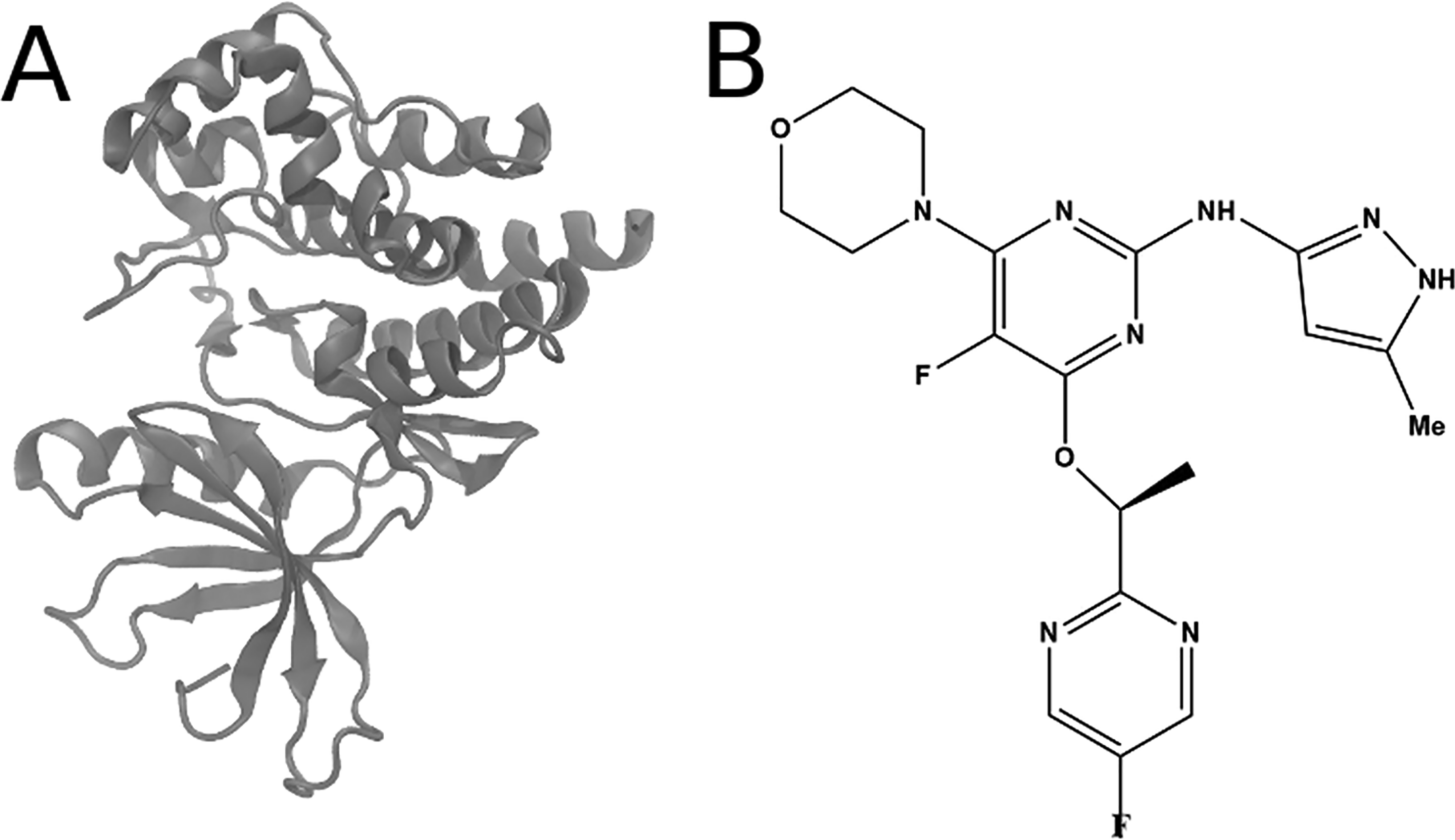
JAK2 protein (A) and its inhibitor, ligand 6 (B).

For the JAK protein in complex with ligand 6,^59^ we generated 22 concentric spherical Voronoi cells, whose milestone radii were chosen to produce adequate sampling and favorable boundary collisions within each cell. Using this setup, we computed the *k*_off_ value (and residence time, by extension) for the JAK inhibitor (Figure 5) in four separate SEEKR runs (with the same settings and starting structures for each anchor), yielding an average *k*_off_ of 4.6 ± 0.1 × 10^–5^ s^–1^ or a residence time of 6.3 ± 0.1 h. The SEEKR-computed residence time is remarkably similar to an experimental residence time of 6.65 h for this system.^59^ The residence time computed with SEEKR and reported here is the average of four separate runs from the same starting structures. The results of the individual runs as well as an examination of calculation sensitivity to different procedures used to compute the starting structures can be found in the Supporting Information.

At this point, let us address the differences between error margins and convergence reported by SEEKR2 calculations. The apparent discrepancy between these two concepts can be clearly observed in Figures 2–4, where SEEKR2 appears to estimate the rate constants with very high precision, but the estimated quantities appear to bear differences from one another. Error margins mainly depend on the shape of distributions of rate matrices, where the likelihood of a matrix is computed based on the statistics, counts, and times of transitions between milestones observed in simulation (the details of this computation may be found in the Supporting Information). In contrast, the convergence of results depends heavily on how well sampled are the relevant states in a simulation. A new user to SEEKR2 may be uncertain when sufficient simulation time has been obtained to compute kinetic and thermodynamic quantities of high quality for their system. In our experience, at least 250 ns, and often as much as 500 or 1000 ns, are needed to obtain converged SEEKR calculations. Once each anchor has this amount of simulation time, one should use converge.py to plot the convergence of *k*_off_, *k*_on_, or Δ*G*_bind_, where convergence may be visually assessed. We believe that it is reasonable to declare a quantity as converged if its fluctuations lie within 10% of its mean value over a significant period of simulation time—for example, 200 ns. One should also confirm that sufficient transitions have occurred between surfaces—ideally at least 100 intersurface transitions.

A system may transit between a series of energy basins, and all of these regions must be sampled thoroughly if good convergence is to be expected. Therefore, one many imagine the hypothetical situation where many transitions between milestones have been observed, but there was sufficient time in the trajectory to sample only one of multiple important energy basins—yielding small error margins but wide convergence errors. SEEKR2 includes additional tools, such as converge.py, that allow users to better analyze the quality of convergence for their system of interest.

We have included a flow chart diagram in the Supporting Information that details the inputs and the main steps of the SEEKR2 computation (Figure S3).

## Conclusions

In summary, SEEKR2 performs MMVT and conventional milestoning simulations with an extensible interface for milestone/Voronoi cell definitions and performs the simulations using either a CPU platform, or, much more quickly and efficiently, using a GPU platform. Only results using one-dimensional concentric spherical milestone shapes have been used for this study, but more dimensions and other milestone shapes have been implemented and will be straightforward to utilize. We have shown that SEEKR2, in general, performs better than earlier versions of SEEKR to recreate the kinetics and thermodynamics for two benchmark systems and has performed well on a new, challenging system with a complex ligand and slow unbinding kinetics. The new HMR feature allows for even faster calculations while still giving correct results for the systems in this study. Additional work will need to further validate the correctness of using HMR in simulations used to compute kinetics and under what circumstances HMR is suitable for that task. SEEKR2 contains many of the utilities that members of the biophysics community may find useful for their own milestoning calculations.

## Materials and Methods

The SEEKR2 calculations in this paper required both MD and BD simulations, which accept different sorts of inputs. The analysis (calculation of kinetic and thermodynamic quantities, error analysis, and convergence analysis) was performed by SEEKR2. When possible, temperatures, salt concentrations, and protonation states were set to recreate experimental conditions as closely as possible. One exception to this was the MD simulations of the β-cyclodextrin “host” with the 1- or 2-naphylethanol “guest”, which had 0.5 M MnSO_4_ dissolved in solution for the experiment.^60^ Due to the inadequacy or lack of parameters for these divalent ions, we elected to use pure water in these MD simulations, as we did with the other host–guest systems, which is consistent with previous studies.^58,61^ We also choose not to add the divalent salts to the BD stage as the use of divalent ions with the Poisson–Boltzmann equation was likely to cause inaccuracies. All Δ*G*_bind_ values were computed using the formula Δ*G*_bind_*= RT* ln(*k*_off_/*k*_on_), where *R* is the gas constant, *T* is the temperature, and ln() is the natural logarithm function.

### Molecular Dynamics Simulations

All MD were performed with OpenMM using the SEEKR2 OpenMM Plugin. Simulations were initiated through the SEEKR2 API. MMVT simulations were performed according to the prescribed procedure.^62^ All starting structures and parameters were reused from the previous SEEKR MMVT study, and the same collective variable definitions, site locations, and concentric spherical milestone shapes were used.^46^

### Brownian Dynamics Simulations

All BD were performed using the Browndye 2 program.^63^ As with OpenMM, all simulations were prepared and controlled through the SEEKR API. Interior dielectrics were set to 4, while exterior dielectric constants were set to 78. All atomic positions, charges, and radii were reused from the previous SEEKR-MMVT study. The APBS program was used to compute electrostatic grids.^64^ Desolvation forces and hydrodynamic interactions were enabled for all calculations, and other physical quantities (such as viscosity, solvent radius, etc.) were left at their defaults.

### Trypsin-Benzamidine System

Simulations of the trypsin-benzamidine system were performed in an almost identical fashion to our previous study, in which we parametrized the protein using the AMBER ff14SB forcefield, and the ligand with Antechamber,^46^ with all simulations performed at 298.15 K and, in the MD simulations, with rigid hydrogen-heavy atom bonds, and a non-bonded cutoff of 9 Å, and a timestep of 2 fs (4 fs for HMR), using OpenMM. The OpenMM implementation allowed us to check for a collision every timestep, instead of every 10 timesteps of the previous implementation,^46^ which likely improved calculation accuracy. We added some additional milestones to the trypsin-benzamidine system such that milestones were located at 1, 2, 3, 4, 6, 8, 10, 12, 14, 16, and 18 Å from the center of mass of the binding site. Starting structures within each Voronoi cell were generated from an SMD simulation, where the system was started from a bound state configuration and pulled out to a site ligand from a distance of 1 Å to a distance of 13 Å over the course of 20 ns of constant volume (NVT) MD using a moving harmonic restraint with a spring constant of 9000 kJ·mol^–1^·nm^–2^. Upon examination, we determined that the original 14 Å milestones showed anomalous results in the BD, probably due to solvation shell effects and steric hindrances caused by the rigid body approach in BD. We added milestones beyond the original 14 Å to improve the calculation by moving the BD region beyond the solvation shells. Starting structures beyond the 14 Å milestone were extracted sequentially from the states generated at the moments of MMVT collisions against lower milestones. Using the generated starting structures for each Voronoi cell, a total of 500 ns of MMVT MD simulations per cell were performed using SEEKR2. All collisions against the milestones were recorded for later analysis. For the BD simulations, the ligand was started at the b-surface and proceeded until it either escaped or satisfied the “reaction criteria” of touching the 18 Å radius milestone (b-surface stage). Then, among those that touched the 18 Å milestone, 1000 structures were extracted, and from each of these, 1000 independent BD simulations were run until the ligands either escaped or touched the 16 Å milestone (BD milestone stage). The purpose of the b-surface stage is to compute the rate of initial encounter with the outermost milestone, while the BD milestone stage computes transition probabilities used in the milestoning model, which are used, in combination with the initial encounter rate, to compute the *k*_on_.

### Host–Guest Systems

For the host–guest systems, all parameters and starting structures were identical to our previous SEEKR papers where we used the Q4MD forcefield for host and guest parametrizations.^46,47^ However, to better recreate experimental conditions, a few adjustments to the host–guest system simulations were necessary. The conditions used to generate the experimental quantities are summarized in Table 3. In previous studies, all SEEKR calculations had been performed at 298.15 K, but in this study, we simulated the 1-naphthyl-ethanol and 2-naphthyl-ethanol guest compounds at 293.15 K to match experiment more closely. As in our previous study, we elected to perform all MD and BD simulations in pure water due to the difficulty of correctly representing divalent electrolytes in MD and also in the Poisson–Boltzmann formulation used in BD.

**Table 3:** Experimental Conditions when Measuring the Kinetics of Binding/Unbinding for the Host–Guest System^a^

guest molecule	experimental study	temperature (K)	salt	pH
1-propanol	Fukahori et al.^66^	298.15	pure water	7
1-butanol	Fukahori et al.^66^	298.15	pure water	7
*tert*-butanol	Fukahori et al.^66^	298.15	pure water	7
1-naphthyl-ethanol	Barros et al.^60^	293.15	0.5 M MnSO_4_	7
2-naphthyl-ethanol	Barros et al.^60^	293.15	0.5 M MnSO_4_	7
methyl butyrate	Nishikawa et al.^67^	298.15	pure water	7
aspirin (protonated)	Fukahori et al.^68^	298.15	pure water	1.7

a We attempted to recreate these conditions as closely as possible in our simulations/calculations.

New SMD simulations were performed where the centers of masses (COMs) of “guest” ligands were restrained to 0.5 Å from the COM of the β-cyclodextrin “host” for 10 ns of constant pressure MD (NPT). Following this, the guest molecules were pulled by a moving harmonic restraint with a spring constant of 90,000 kJ·mol^–1^·nm^–2^ in an SMD simulation from the 0.5 Å starting location to a final COM-COM distance of 13.5 over the course of 100 ns of NVT MD. The purpose of the SMD simulations was to generate starting structures between each pair of milestones (near the center of each Voronoi cell). Using these starting structures for the MD simulations, MMVT simulations were run using SEEKR for 50 ns per Voronoi cell, which is equal to the maximum simulation length per anchor of the previous MMVT study we performed.^46^ BD simulations of both the b-surface stage and the BD milestone stage were run to compute host–guest *k*_on_s.

### JAK Systems

The starting structure was obtained from the X-ray crystal structure of the JAK2-inhibitor complex domain with PDB ID: 3ZMM. Forcefield parameterization was done using the AMBER ff14SB forcefield with explicit solvation in a truncated octahedron 10 Å periodic box, 150 mM salt concentration, and a non-bonded cut-off radius of 9 Å. The ligand was parametrized using Antechamber in AmberTools with partial charges assigned by the AM1-BCC semiempirical charge model and all other parameters assigned from GAFF. The solvated complex was slowly heated to 300 K followed by 20 ns each of NPT and NVT MD preparatory simulation carried out at 300 K. All MD simulations have been carried out using the OpenMM simulation engine. SMD simulations were then performed where the COM of the inhibitor was restrained to 2.5 Å from the COM of the binding region of the receptor for 20 ns of constant pressure MD (NPT). Subsequently, the inhibitor was pulled by a moving harmonic restraint with a spring constant of 50,000 kJ·mol^–1^·nm^–2^ in an SMD simulation from the 2.5 Å starting location to a final COM-COM distance of 16.0 Å over the course of 1000 ns of NVT MD. Concentric spherical Voronoi cells or “milestones” were defined at distances of 2.5, 3.0, 3.5, 4.0, 4.5, 5.0, 5.5, 6.0, 6.5, 7.0, 7.5, 8.0, 8.5, 9.0, 9.5, 10.0, 11.0, 12.0, 13.0, 14.0, 15.0, and 16.0 Å from the COM of the binding site. MMVT simulations were then run using SEEKR for 400 ns per Voronoi cell.

## Supporting Information Available

Additional data, figures, tables, and equations including a formulation of MMVT error analysis, convergence of the *k*_off_ and *k*_on_ for the trypsin-benzamidine system, the workflow diagram of the SEEKR2 procedure, and results of four separate runs of the JAK system can be found in the SI (PDF)

## Notes

SEEKR2 can be found at https://github.com/seekrcentral/seekr2.git, which also includes documentation about installation instructions and several tutorials for how to prepare and use SEEKR2 to compute the kinetics and thermodynamics of molecular systems of interest. The SEEKR2 OpenMM plugin can be found at https://github.com/seekrcentral/seekr2_openmm_plugin.git. The data for this study can be obtained at https://doi.org/10.6075/J0668DDR.

## ABBREVIATIONS

SEEKR2: simulation-enabled estimation of kinetic rates version 2
MMVT: Markovian milestoning with Voronoi tessellations
MD: molecular dynamics
BD: Brownian dynamics
COM: center of mass
NVT: constant number of particles, volume, and temperature simulation
NPT: constant number of particles, pressure, and temperature simulation
API: application programming interface
