# Supplementary material for: SEEKR2: Versatile Multiscale Milestoning Utilizing the OpenMM Molecular Dynamics Engine
Source: J Chem Inf Model. 2022 Jun 27;62(13):3253–62. doi: 10.1021/acs.jcim.2c00501 (PMC9277580; doi:10.1021/acs.jcim.2c00501)
Supplement: Supplementary file 1 — ci2c00501_si_001.pdf [file ci2c00501_si_001.pdf]

SEEKR2: Versatile Multiscale Milestoning Utilizing the OpenMM Molecular Dynamics Engine

Supplementary Information

*Lane W. Votapka, Andrew M. Stokely, Anupam A. Ojha, Rommie E. Amaro**

University of California, San Diego, 9500 Gilman Dr. La Jolla, CA 92093

S1. Error Analysis of MMVT Simulations

Elber's Original Milestoning Error Analysis

A foundational paper[1] contains useful information about formulating the distribution of rate matrices Q if conventional milestoning quantities N_{ij} and R_i (the "data") are observed directly from the simulation trajectories (such as in Elber's original milestoning formulations). Given this "posterior" conditional probability distribution $p(Q|data)$, one may compute a distribution of all resulting kinetics and thermodynamics quantities computed using Q. By obtaining the standard deviations of these distributions, one may obtain error margins for computed thermodynamics and kinetics quantities.

Finding $p(Q|data)$ involves a Bayes' rule formulation:

$$p(Q \mid data) = \frac{p(data|Q) \cdot p(Q)}{p(data)} \qquad \text{Eq. S1}$$

The so-called "likelihood" $p(data|Q)$ and "prior" $p(Q)$ probability distributions can be estimated using a Monte Carlo approach, as we outlined in an earlier SEEKR paper.[2] The marginalization $p(data)$ is assumed to be a uniform distribution, so we assume that the posterior is proportional to the numerator of Eq. S1.

We now need an expression for the likelihood and prior distributions, which is explicitly provided for the original milestoning approach proposed by Elber.[1,3] However, since we have been unable to find a formal expression in the literature for the likelihood and prior in calculations using MMVT simulations, we develop an MMVT error estimation approach later in this section.

For now, we revisit the error estimation approach used for Elber's original milestoning formulation. Given an observed trajectory where a series of milestones $i(t')$ are visited at different times t', one may

construct a probability distribution $\rho_{ij}(t)$ that the trajectory will travel from state i to state j with an incubation time of exactly t.

$$\rho_{ij}(t) = q_{ij} e^{\sum_{j \neq i} q_{ij} t} \quad \text{Eq. S2}$$

For a given rate matrix Q, the probability of an observed trajectory visiting the simulated sequence of states $i_1(t_1)$, $i_2(t_2)$, $i_3(t_3)$, … becomes

$$\rho_{i_0 i_1}(t_1) \rho_{i_1 i_2}(t_2 - t_1) \rho_{i_2 i_3}(t_3 - t_2) ... \quad \text{Eq. S3}$$

for the entire sequence of states $i(t')$ visited. Combining Eqs. S2 and S3, Vanden-Eijnden et al.[3] reorganizes them to produce the likelihood of observing the simulated trajectory (the data) given a definition of the rate matrix Q.

$$p(data \,|\, Q) = \prod_{i=1}^{N} \prod_{j \neq i} q_{ij}^{N_{ij}} e^{-q_{ij} R_i} \quad \text{Eq. S4}$$

In a later paper, Majek et al.[1] uses Bayesian reasoning similar to Eq. S1 to compute the probability of a rate matrix given the simulation data.

$$p(Q \,|\, data) \propto \prod_{i=1}^{N} \prod_{j \neq i} q_{ij}^{N_{ij}} e^{-q_{ij} R_i} p(Q) \quad \text{Eq. S5}$$

In a previous paper of ours[2] we outline a Monte Carlo algorithm for sampling matrices from the distribution in Eq. S5.

MMVT Error Analysis

In the original milestoning formulations by Elber et al.,[1,4–7] the quantities N_{ij} and R_i are observed directly from the trajectories. However, in MMVT, they are not directly observed by the trajectories, but are rather computed as intermediate quantities from the following equations:

$$N_{ij} = T \sum_{\alpha=1}^{\Lambda} \pi_\alpha \frac{N_{ij}^{\alpha}}{T_\alpha} \quad \text{Eq. S6}$$

$$R_i = T \sum_{\alpha=1}^{\Lambda} \pi_\alpha \frac{R_i^{\alpha}}{T_\alpha} \quad \text{Eq. S7}$$

where,

$$T = \left(\sum_{\alpha=1}^{\Lambda} \frac{\pi_\alpha}{T_\alpha} \right)^{-1} \quad \text{Eq. S8}$$

$$\sum_{\beta=1}^{\Lambda} \pi_\beta k_{\beta,\alpha} = \sum_{\substack{\beta=1 \\ \beta \neq \alpha}}^{\Lambda} \pi_\alpha k_{\alpha,\beta}, \sum_{\alpha=1}^{\Lambda} \pi_\alpha = 1 \qquad \text{Eq. S9}$$

$$k_{\alpha,\beta} = \frac{N_{\alpha,\beta}}{T_\alpha} \qquad \text{Eq. S10}$$

Where all MMVT simulations are constrained to remain within an anchor α, T_α is the total simulation time spent in anchor α, $N_{\alpha,\beta}$ is the count of the total number of times a simulation within anchor α collided with the boundary (milestone) between anchor α and anchor β, N_{ij}^{α} is the count of the number of times a simulation in anchor α had first collided with milestone i and had travelled across the anchor to collide with some different anchor j ($j \neq i$), and R_i^{α} is the total time spent in the simulation of anchor α after having collided with milestone i, but before colliding with any other milestone. The stationary probability distribution for the anchors $\boldsymbol{\pi}$ is also computed as an intermediate quantity.

Unfortunately, the formulation in Eq. S5 and the Monte Carlo algorithm, as they are, break down with MMVT, since in MMVT the N_{ij} and R_i quantities obtained from Eqs. S6 and S7 do not actually represent the number of transitions or incubation times directly observed in the data, and therefore cannot be placed into Eqs. S4 or S5. Trying to do so would cause incorrect $p(data|Q)$ distributions when using the MMVT procedure.

Therefore, in order to obtain the correct distribution $p(data|Q)$ from the quantities directly sampled in MMVT, let us employ the following approach.

As in Vanden-Eijnden et al.,[3] let us consider a long ergodic trajectory (or more properly, a set of trajectories) that is constrained to remain within an anchor α, and periodically collides with boundaries β_1, β_2, … adjacent to other anchors. The probability $\rho_{\alpha,\beta}(t)$ that the trajectory in anchor α bounces against the milestone adjacent to anchor β after spending time t since colliding with any boundary is

$$\rho_{\alpha,\beta}(t) = k_{\alpha,\beta} e^{\sum_{\alpha \neq \beta} k_{\alpha,\beta} t} \qquad \text{Eq. S11}$$

For a given flux matrix K, the probability of an observed trajectory visiting the simulated sequence of bounced boundaries $\beta_1(t_1)$, $\beta_2(t_2)$, $\beta_3(t_3)$, … becomes

$$\rho_{\beta_0\beta_1}(t_1)\rho_{\beta_1\beta_2}(t_2 - t_1)\rho_{\beta_2\beta_3}(t_3 - t_2)... \qquad \text{Eq. S12}$$

This gives us a likelihood distribution of observing a set of data given a flux matrix K.

$$p(data \mid K) = \prod_{\alpha=1}^{\Lambda} \prod_{\alpha \neq \beta} k_{\alpha,\beta}^{N_{\alpha,\beta}} e^{-k_{\alpha,\beta} T_\alpha} \qquad \text{Eq. S13}$$

We may then use the same Bayesian reasoning employed in Eq. S1.

$$p(K|data) \propto \prod_{\alpha=1}^{\Lambda} \prod_{\alpha \neq \beta} k_{\alpha,\beta}^{N_{\alpha,\beta}} e^{-k_{\alpha,\beta} T_\alpha} \cdot p(K) \qquad \text{Eq. S14}$$

This may be used to sample a distribution of flux matrices *K* conditional on the data, which can then sample a distribution of stationary probabilities across the anchors $\boldsymbol{\pi}$.

Next, we must estimate the likelihood of a set of transitions between a series of milestones i_1, i_2, i_3, … in a continuous-time Markov jump process, but that is constrained to remain within an anchor α. A reduced description of this trajectory may be represented by the piecewise continuous function *i(t)*, which represents the index of the last milestone touched at time *t* (while constrained to remain in anchor α).

As mentioned in Vanden-Eijnden et al.,[3] given that at a time *t* the trajectory is in state *i* (last touched milestone *i*), the probability that it stays in state *i* until time $t' \geq t$ then jumps to state $j \neq i$ in the time interval $[t', t' + \Delta t]$ is, to leading order in *t*, given by

$$\rho_{ij}^{\alpha}(t) = q_{ij}^{\alpha} e^{\sum_{j \neq i} q_{ij}^{\alpha} t} \qquad \text{Eq. S15}$$

where $q_{ij}^{\alpha} = N_{ij}^{\alpha}/R_i^{\alpha}$.

As before, we may estimate the probability of observing a particular set of data given the observed quantities.

$$\rho_{i_0 i_1}^{\alpha}(t_1)\rho_{i_1 i_2}^{\alpha}(t_2 - t_1)\rho_{i_2 i_3}^{\alpha}(t_3 - t_2)... \qquad \text{Eq. S16}$$

As before, we may obtain the likelihood distribution, and then the posterior.

$$p(data \,|\, Q^{\alpha}) = \prod_{i=1}^{N_{\alpha}} \prod_{j \neq i} q_{ij}^{\alpha N_{ij}^{\alpha}} e^{-q_{ij}^{\alpha} R_i^{\alpha}} \qquad \text{Eq. S17}$$

$$p(Q^{\alpha} \,|\, data) \propto \prod_{i=1}^{N_{\alpha}} \prod_{j \neq i} q_{ij}^{\alpha N_{ij}^{\alpha}} e^{-q_{ij}^{\alpha} R_i^{\alpha}} \cdot p(Q^{\alpha}) \qquad \text{Eq. S18}$$

Where $N_{\alpha} = \sum_{\beta} N_{\alpha,\beta}$. Given Eq. S18, we may sample matrices Q^{α} and reconstruct the necessary quantities to compute *Q* by realizing $N_{ij}^{\alpha *} = q_{ij}^{\alpha} \cdot R_i^{\alpha}$, where $N_{ij}^{\alpha *}$ is a quantity to enter into Eq. S6 that is not observed in the data, but is sampled from the likelihood distribution. This is, admittedly, strange, because this will result in non-integer values for $N_{ij}^{\alpha *}$. Nevertheless, by sampling Eqs. S14 and S18, one may obtain all quantities needed to complete an MMVT calculation and obtain an estimate for the thermodynamics and kinetics quantities.

By extracting large numbers (hundreds or thousands) of matrices from the distributions in Eqs. S14 and S18, and by them performing the MMVT milestoning procedures on each of them to compute distributions of the desired kinetics and thermodynamics results, one may compute the error margins for each of the results by finding the standard deviations for each of the distributions.

Monte Carlo Algorithm for Sampling MMVT Matrices

We use a nonreversible element shift Monte Carlo algorithm to sample the posterior probability in Eqs. S14 and S18, which was inspired by algorithms developed for Markov state models[8] and the algorithm developed by us to use for Elber's original milestoning formulation.[2]

To sample the matrices in Eqs. S14 and S18, we use a Metropolis criterion which determines whether we accept a proposed matrix in the available space. Since their distribution equations are so similar, we will

use Q as a generic symbol to represent either K in Eq. S14 or Q^{α} in Eq. S18, and an essentially identical procedure may be used to sample them.

Given a proposed matrix Q' and a current matrix Q, the probability that we accept Q' as a member of the distribution is

$$p_{accept} = \frac{p(Q'|data)p(Q')}{p(Q|data)p(Q)} \qquad \text{Eq. S19}$$

A proposed change Δ relates the difference between an element of Q and Q'.

$$q'_{ij} = q_{ij} + \Delta \qquad \text{Eq. S20}$$
$$q'_{ii} = q_{ii} - \Delta$$

Note that the proposed change must ensure that all non-diagonal entries remain positive, and that the main diagonal elements remain the negative sum of the off-diagonal row entries of Q'. To maintain these requirements, the change Δ is drawn from an exponential distribution on the range

$$\Delta \in [-q_{ij}, \infty) \qquad \text{Eq. S21}$$

Note that the mean value is at zero. This also provides a definition for the ratio of prior distributions in Eq. S19.

$$\frac{p(Q')}{p(Q)} = \frac{\prod_{i,j} e^{-q'_{ij}}}{\prod_{i,j} e^{-q_{ij}}} \qquad \text{Eq. S22}$$

We then obtain a full expression of the acceptance probabilities for both K and Q^{α}, assuming that only a single element of the matrix is changed at a time.

$$p_{accept} = \frac{p(K'|data)p(K')}{p(K|data)p(K)} = \left(\frac{k_{\alpha,\beta} + \Delta}{k_{\alpha,\beta}}\right)^{N_{\alpha,\beta}} \frac{e^{-(k_{\alpha,\beta} + \Delta)T_{\alpha}}}{e^{-(k_{\alpha,\beta})T_{\alpha}}} \cdot \frac{e^{-(k_{\alpha,\beta} + \Delta)}}{e^{-(k_{\alpha,\beta})}} \qquad \text{Eq. S23}$$

$$p_{accept} = \frac{p(Q^{\alpha'}|data)p(Q^{\alpha'})}{p(Q^{\alpha}|data)p(Q^{\alpha})} = \left(\frac{q^{\alpha}_{ij} + \Delta}{q^{\alpha}_{ij}}\right)^{N^{\alpha}_{ij}} \frac{e^{-(q^{\alpha}_{ij} + \Delta)R^{\alpha}_{i}}}{e^{-q^{\alpha}_{ij}R^{\alpha}_{i}}} \cdot \frac{e^{-(q^{\alpha}_{ij} + \Delta)}}{e^{-q^{\alpha}_{ij}}} \qquad \text{Eq. S24}$$

S2. Convergence of Systems

In SEEKR2, convergence calculations are performed upon request in a highly automated fashion. Convergence plots of the k_{off} (Figure S2) and the k_{on} (Figure S3) of the trypsin-benzamidine system are shown below as proof of convergence and a demonstration of this capability.

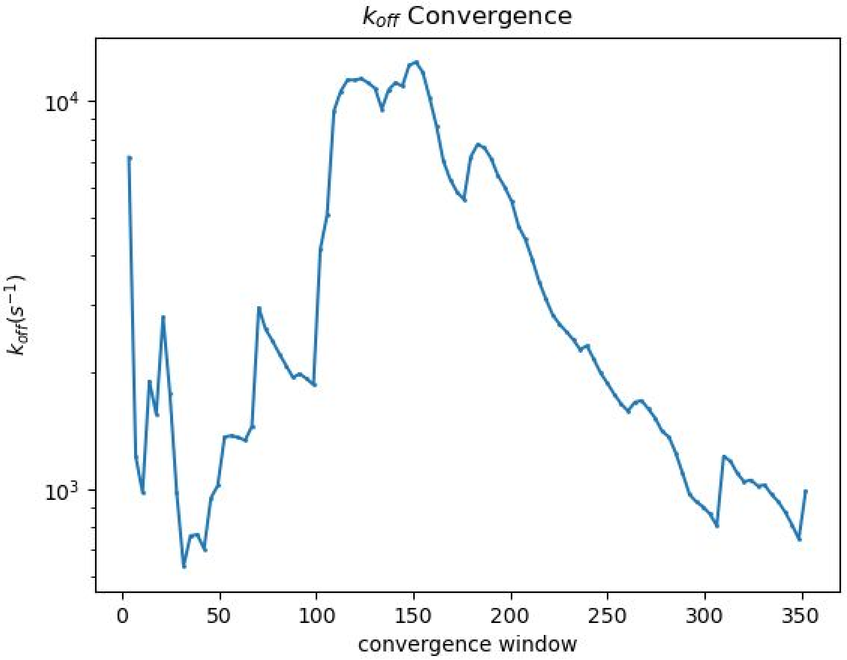

Figure S1: The convergence of the k_{off} for the trypsin/benzamidine system is plotted. The convergence window (x-axis) represents the size of the window of transition data extracted, going from the first few transitions recorded (near the left of the figure) to the full span of transition data observed in each Voronoi cell (near the right of the figure). While the k_{off} still appears to be fluctuating, even by the end of the simulations, notice that the fluctuations have remained approximately within the same order of magnitude of the final k_{off} value. While it is possible that the k_{off} will change significantly with more simulation time, it is not very likely.

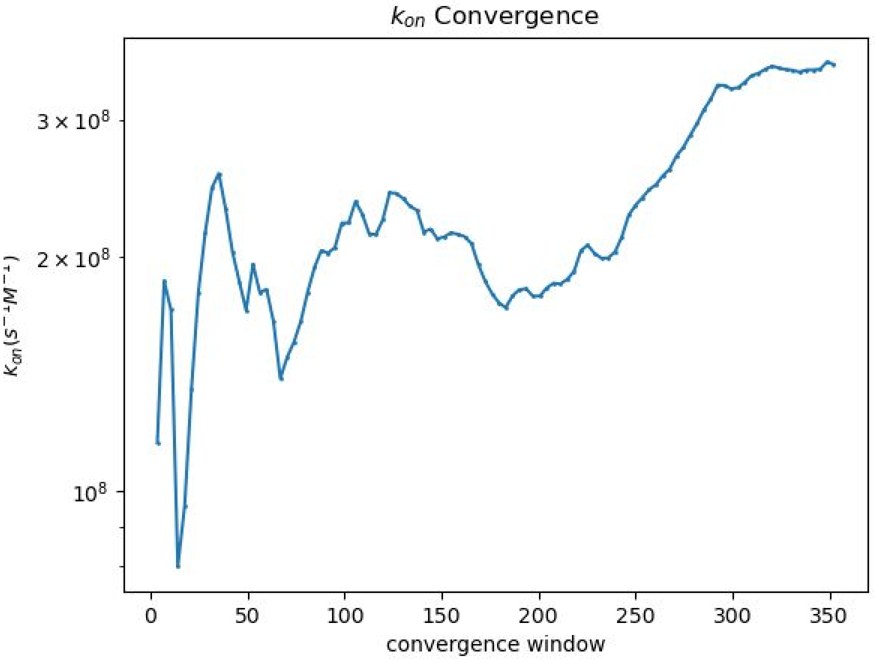

Figure S2: The convergence of the k_{on} for the trypsin/benzamidine system is shown here. The convergence window is based exclusively on the fraction of transition statistics obtained from MD simulations, not BD. Based on the magnitude of fluctuations of the k_{on} in comparison to the magnitude of the k_{on} value itself, this quantity appears converged – more MD simulations are unlikely to change this value significantly.

S3. JAK Detailed System Results

The results for JAK reported in the main text were the average of four separate runs performed from the same starting conformations generated by 1000 ns of steered molecular dynamics (SMD), and otherwise identical settings, whose results can be seen in Table S1. All four kinetics results fall within a close range of one another – well within an order of magnitude. The closeness of all four runs' kinetics bodes well for SEEKR2's reproducibility.

Run	Residence time (hours)	k_{off} (s^{-1})
1	5.8±0.3	$4.8 \pm 0.2 \times 10^{-5}$
2	6.6±0.3	$4.2 \pm 0.2 \times 10^{-5}$
3	4.7±0.2	$5.9 \pm 0.3 \times 10^{-5}$
4	8.0±0.5	$3.5 \pm 0.2 \times 10^{-5}$
**Avg.**	**6.3±0.1**	$\mathbf{4.6 \pm 0.1 \times 10^{-5}}$

Table S1: The results of four separate runs of the JAK system with its inhibitor are shown. The average values are the quantities reported in the main text.

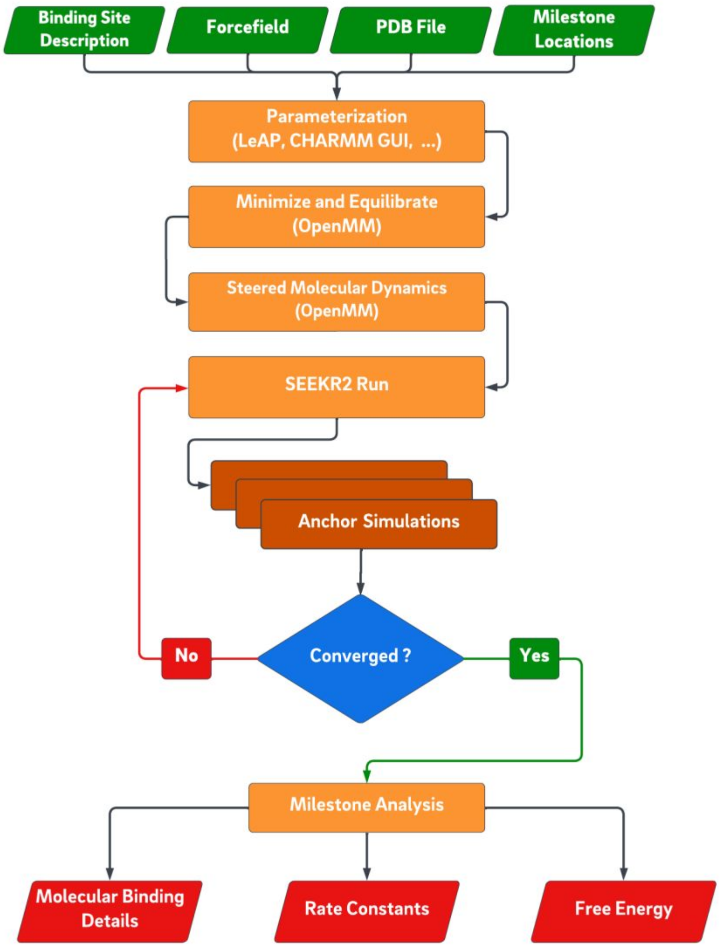

Figure S3: A workflow diagram of the SEEKR2 procedure is shown. Inputs and outputs are represented with green and red parallelograms, respectively. Serial operations are represented in light orange rectangles, while parallel operations, such as the simulations of each anchor, are represented as dark orange rectangles. One conditional branch for whether the SEEKR2 calculation is sufficiently converged is represented as a blue diamond.

References

(1) Májek, P.; Elber, R. Milestoning without a Reaction Coordinate. *J. Chem. Theory Comput.* **2010**, *6* (6), 1805–1817. https://doi.org/10.1021/ct100114j.

(2) Votapka, L. W.; Amaro, R. E. Multiscale Estimation of Binding Kinetics Using Brownian Dynamics, Molecular Dynamics and Milestoning. *PLOS Comput. Biol.* **2015**, *11* (10), e1004381. https://doi.org/10.1371/journal.pcbi.1004381.

(3) Vanden-Eijnden, E.; Venturoli, M. Markovian Milestoning with Voronoi Tessellations. *J. Chem. Phys.* **2009**, *130* (19), 194101. https://doi.org/10.1063/1.3129843.

(4) Faradjian, A. K.; Elber, R. Computing Time Scales from Reaction Coordinates by Milestoning. *J. Chem. Phys.* **2004**, *120* (23), 10880–10889. https://doi.org/10.1063/1.1738640.

(5) West, A. M. A.; Elber, R.; Shalloway, D. Extending Molecular Dynamics Time Scales with Milestoning: Example of Complex Kinetics in a Solvated Peptide. *J. Chem. Phys.* **2007**, *126* (14), 145104. https://doi.org/10.1063/1.2716389.

(6) Bello-Rivas, J. M.; Elber, R. Exact Milestoning. *J. Chem. Phys.* **2015**, *142* (9), 94102. https://doi.org/10.1063/1.4913399.

(7) Vanden-Eijnden, E.; Venturoli, M.; Ciccotti, G.; Elber, R. On the Assumptions Underlying Milestoning. *J. Chem. Phys.* **2008**, *129* (17), 174102. https://doi.org/10.1063/1.2996509.

(8) Noé, F. Probability Distributions of Molecular Observables Computed from Markov Models. *J. Chem. Phys.* **2008**, *128*, 244103. https://doi.org/10.1063/1.2916718.
